# Supplementary material for: DeepAction: a MATLAB toolbox for automated classification of animal behavior in video
Source: Sci Rep. 2023 Feb 15;13:2688. doi: 10.1038/s41598-023-29574-0 (PMC9932075; doi:10.1038/s41598-023-29574-0)
Supplement: Supplementary file 1 — Supplementary Information. [file 41598_2023_29574_MOESM1_ESM.docx]

**Extended data figures**


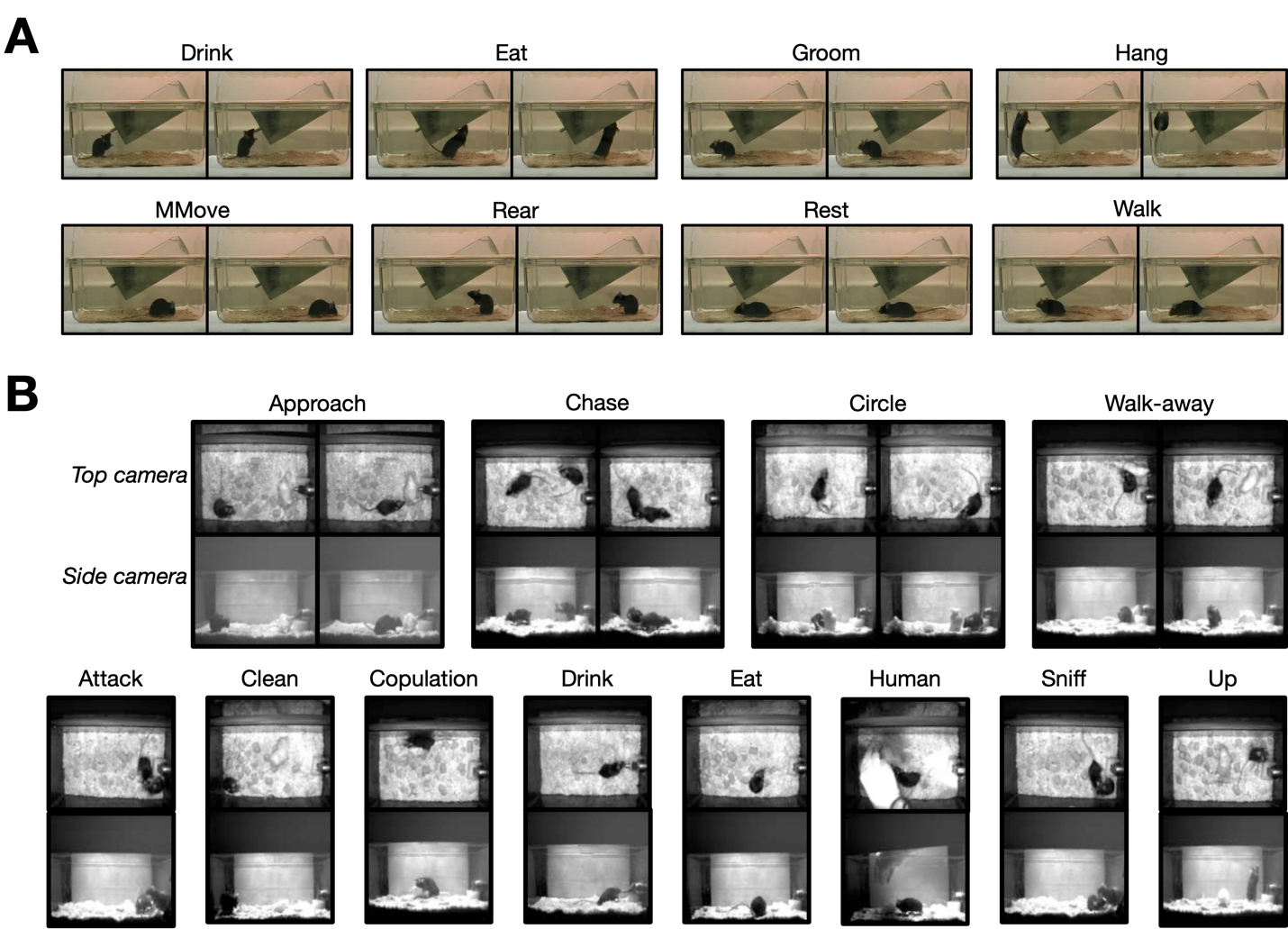


**Figure S1. Example behaviors.** (**A**) Examples of the eight behaviors included in the home-cage dataset. (**B**) Examples of the 12 social behaviors of interest in the CRIM13 database (“other” not shown). Screenshots in (**B**) from Burgos-Artizzu, et al. ^6^.


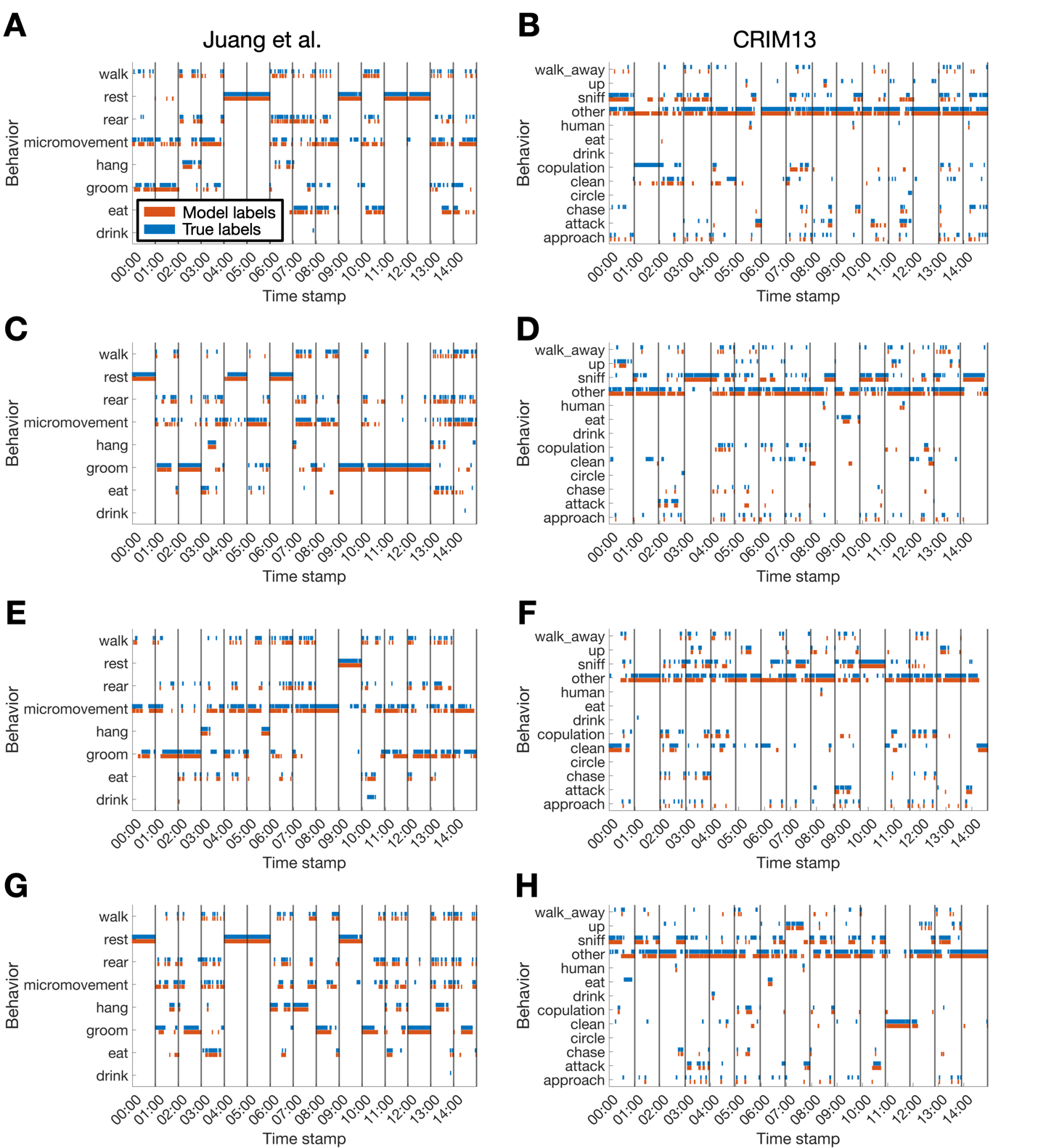


**Figure S2. Sample ethograms with different training data proportions.** Plotted are ethograms corresponding to 20 minutes of randomly-selected clips with (**A,B**) 10, (**C,D**) 20, (**E,F**) 50, and (**G,H**) 80 percent of data used to train the classifier. Black lines denote the start of a new clip, and the data between the black lines denotes the ethogram corresponding to that clip.

**
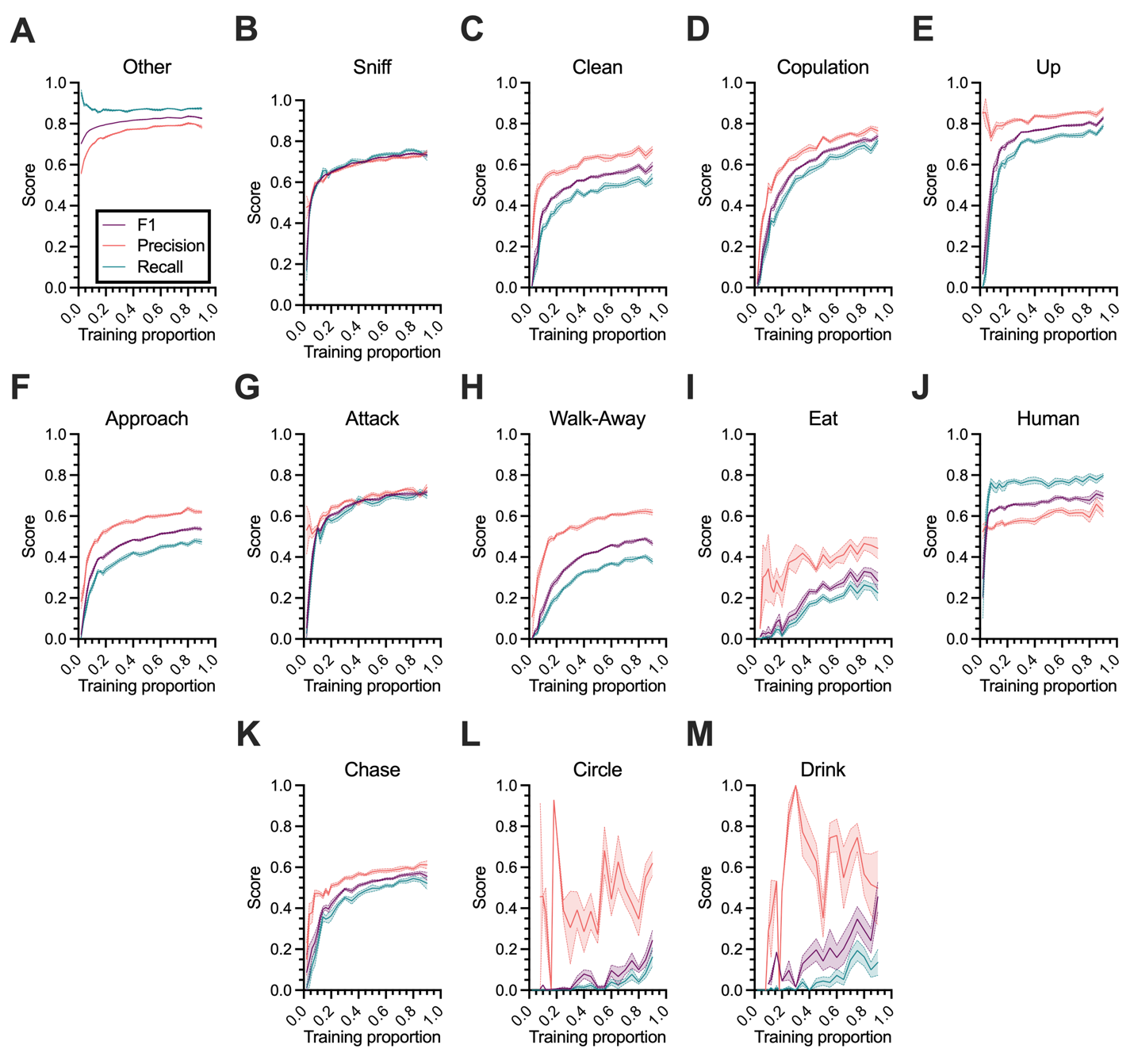
**

**Figure S3. CRIM13 classifier performance by behavior.** Precision, recall, and F1 scores by behavior as a function of the proportion of data used to train the classifier on the CRIM13 dataset. Lines and shaded regions indicate mean and standard error, respectively, across 10 random splits of the data.


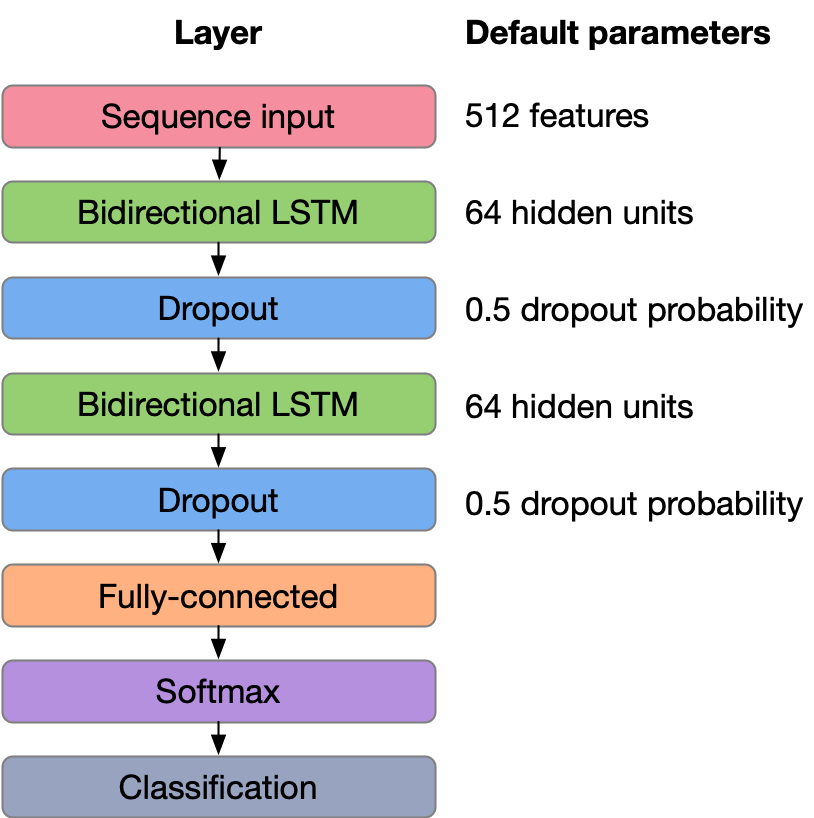


**Figure S4. Schematic of the classification model.** In our analysis, we use a recurrent neural network that accepts input sequences with a dimensionality of $512$. The sequence input layer is followed by a pair of bidirectional LSTM ($64$ hidden units) and dropout (with probability $0.50$) layers. A fully connected layer accepts output from the second dropout layer and is followed by a softmax layer and a sequence-to-sequence classification layer, which returns the final output (i.e., a set of behavioral labels corresponding to each frame in the input).
